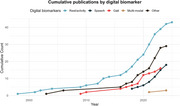# Digital Biomarkers in Early Alzheimer's Disease from Wearables or Portables: A Scoping Review

**DOI:** 10.1002/alz70856_102566

**Published:** 2025-12-25

**Authors:** Mathias Holsey Gramkow, Helena Sophia Gleerup, Anja Hviid Simonsen, Gunhild Waldemar, Kristian Steen Frederiksen

**Affiliations:** ^1^ Danish Dementia Research Centre, Dept. of Neurology, Copenhagen University Hospital ‐ Rigshospitalet, Copenhagen, Denmark; ^2^ Danish Dementia Research Centre, Dept. of Neurology, Copenhagen University Hospital ‐ Rigshospitalet, Copenhagen, Denmark, Denmark; ^3^ Department of Clinical Medicine, Faculty of Health and Medical Sciences, University of Copenhagen, Copenhagen, Denmark

## Abstract

**Background:**

Alzheimer's disease (AD) poses significant challenges to healthcare systems, especially with the emergence of disease‐modifying treatments which is expected to increase referrals to memory clinics. The pursuit of accurate biomarkers for early detection and disease management has driven growing interest in digital biomarkers—quantifiable measures obtained through digital health technologies (DHTs). There is limited synthesis of knowledge regarding methods, applications, and gaps in digital biomarker research for AD. This scoping review aims to map the research landscape of digital biomarkers in the early AD population obtained with wearables or portables.

**Method:**

Eligible studies included original research on portable or wearable DHTs assessing non‐cognitive digital biomarkers in adults with early AD (mild cognitive impairment (MCI) or mild dementia) according to established diagnostic criteria. Three databases (MEDLINE, Web of Science, and EMBASE) were searched using a wide search strategy. Screening was conducted using Covidence software, with independent review and data extraction by two review team members.

**Result:**

After de‐duplication, 8893 references were screened for titles and abstracts and 433 for full‐text, resulting in 109 studies that were included in the review. The studies described a wide variety of wearable/portable obtained digital biomarkers in a population of 3462 individuals with MCI due to AD (54.6 % female, weighted mean age 74 years), and 3387 individuals with mild AD (48 % female, mean age 73 years). Most studies described results on rest/activity (39 %), speech (17 %) and graphomotor function (12 %), with most studies focusing on one biomarker domain (Figure 1). Only few studies reported outcomes associated with diagnosis (16 %) and prognosis (3 %), and only one study reported on digital biomarkers for the assessment of treatment monitoring.

**Conclusion:**

We identified a growing source of studies investigating digital biomarkers in early AD, the only population considered eligible for anti‐amyloid therapy. There was a paucity of studies examining the diagnostic and prognostic usefulness of digital biomarkers, which remains a knowledge gap in this population. Our study provides an overview to guide future research efforts to bridge the gap between development and clinical application of novel digital biomarkers in early Alzheimer's disease.